# Ocular manifestations as first signs of systemic T cell lymphoma in two cases

**DOI:** 10.1186/s12886-017-0494-3

**Published:** 2017-06-23

**Authors:** Xiao Zhang, Xin-Shu Liu, Chan Zhao, Ya-Min Lai, Mei-Fen Zhang

**Affiliations:** 1 Department of Ophthalmology, Peking Union Medical College Hospital, Peking Union Medical College, Chinese Academy of Medical Sciences, Beijing, 100730 China; 2 Department of Gastroenterology, Peking Union Medical College Hospital, Peking Union Medical College, Chinese Academy of Medical Sciences, Beijing, 100730 China

**Keywords:** Uveitis, T cell non-Hodgkin’s lymphoma, Type II enteropathy-associated T-cell lymphoma, Case report

## Abstract

**Background:**

Intraocular involvement of systemic T-cell lymphomas are uncommon and have been broadly regarded as markers of poor prognosis. We reported two cases of uveitis patients finally diagnosed as systemic T cell lymphoma.

**Case presentation:**

Case one is a 19-year-old female presented with fever and liver dysfunction, and was diagnosed as EBV-associated chronic active hepatitis. Fourteen months later, she suffered from recurrent granulomatous anterior uveitis in both eyes, which failed to respond to steroid and immunosuppressant therapy. A mass on the left side of pharynx was found and the final diagnosis was pharynx T cell non-Hodgkin’s lymphoma. After 13 cycles of chemotherapy, her systematic symptoms and uveitis relieved a lot, and eye condition is stable after cataract surgery. Case two is a 37-year-old male complaining bilateral blurred vision and recurrent abdominal pain. Panuveitis was diagnosed and anterior inflammation did not release after topical steroid. During the following days, the patient complained intermittent abdominal pain and fever, with rapidly progressive bilateral visual decrease. Final diagnosis was gallbladder type II enteropathy-associated T-cell lymphoma. The patient died of multiple organ failure 4 days after operation that was only 26 days after presenting to our hospital.

**Conclusions:**

Ocular manifestations as first signs of systemic T cell lymphoma were rare. Diagnosis of lymphoma has to be suspected when patients have systemic manifestations including fever, fatigue, abdominal pain, EBV-associated liver disease, et al., and uveitis fails to respond to steroid therapy.

## Background

Intraocular manifestations of systemic T cell lymphoma are rare. Systemic lymphomas usually metastasize though blood into the uveal tissues [1]. Ocular manifestations include vitritis, posterior uveitis with distinct yellowish subretinal epithelium infiltrates and occasionally anterior uveitis and optic nerve involvement [2]. Here we reported two cases of uveitis patients finally diagnosed as systemic T cell lymphoma.

## Case presentation

### Case one

**Fig. 1:**
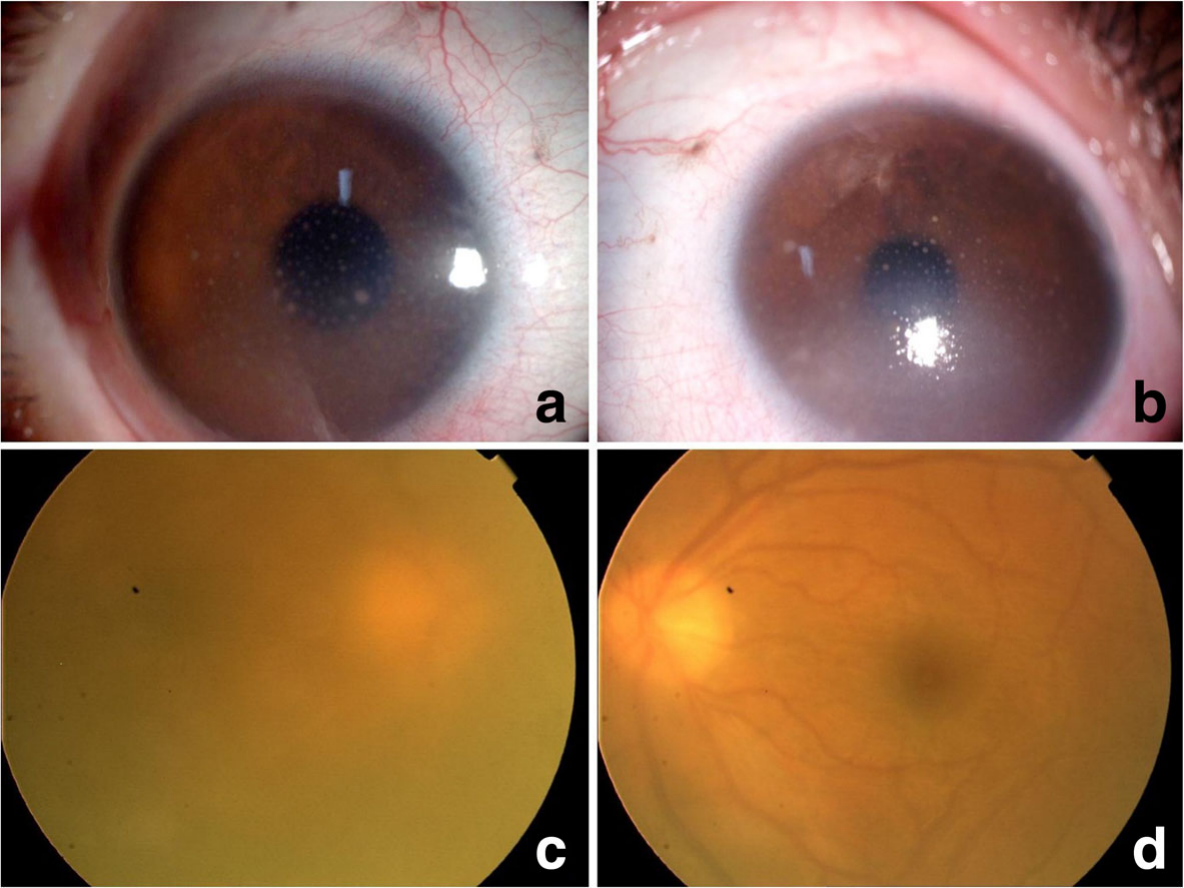
Anterior segment examination of case one (**a**, OD; **b**, OS) showed mixed congestion and diffused mutton-fat KPs; Fundus examination (**c**, OD; **d**, OS) was not clear, but almost normal

**Fig. 2:**
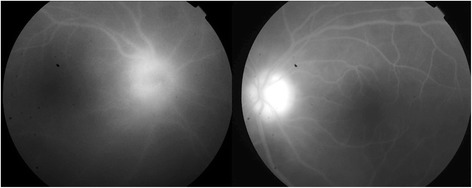
Fundus fluorescein angiography (FFA) of case one showed high fluorescence of optic disk OU

A 19-year-old Chinese female presented in Oct 2005 with fever, fatigue, cough and sputum. Accessory examination revealed positive serum Epstein-Barr virus (EBV) IgG/VCA and IgM/VCA, abnormal hepatic function and erythrocyte sedimentation rate. She was diagnosed as chronic active hepatitis and her symptoms relieved a lot after antibiotics and liver protection treatment. In Dec 2006, she complained of blurred vision and red eye OU, tinnitus and hearing loss of the right ear, as well as rhinobyon. Visual acuity was 20/100, Jr2 OD and 20/250, Jr1, OS, and best corrected visual acuity (BCVA) was not detected at this time. There were mixed congestion, diffused mutton-fat KPs, and anterior chamber cells and flare in both eyes (Fig. 1a, b). Fundus examination was not clear, but normal (Fig. 1c, d). B-scan ultrasonography showed slight vitreous opacities, OU. The diagnosis was “granulomatous uveitis OU”. Anterior uveitis did not respond well to topical 1% Pred Forte, so systemic corticosteroid and azathioprine were given. There were some improvement of anterior segment inflammation after treatment and BCVA improved to 18/20 OD and 20/20 OS at one time. But anterior segment inflammation was persistent and repeated, and cataract developed gradually. Fundus fluorescein angiography (FFA) was performed on Feb 9, 2007, there was high fluorescence of optic disk OU, no obvious fluorescein leakage of the vessels and no macular edema (Fig. 2). In May 2007, she complained of feet pain, lower extremity weakness and intermittent twitch. ENT consultation found a mass on the left side of pharynx. Final diagnosis was pharynx T cell non-Hodgkin’s lymphoma with CD3 (+) and CD56 (−). PCR detected TCR gene rearrangement. Cerebrospinal fluid examination also found massive naive cells. After chemotherapy of CHOP (CHOP = cyclophosphamide, epirulbicin hydrochloride, vindesine sulfate, prednisone) and COID (COID = methotrexate, vindesine sulfate, ifosfamide, dexamethasone) for 13 cycles, fever and feet pain relieved a lot. Phaco and IOL implantation was done for both eyes. Visual acuity improved to 20/16 OD and 20/12.5 OS. So far, her condition is stable for more than 10 years and there is no sign of disease progression.

### Case two

**Fig. 3:**
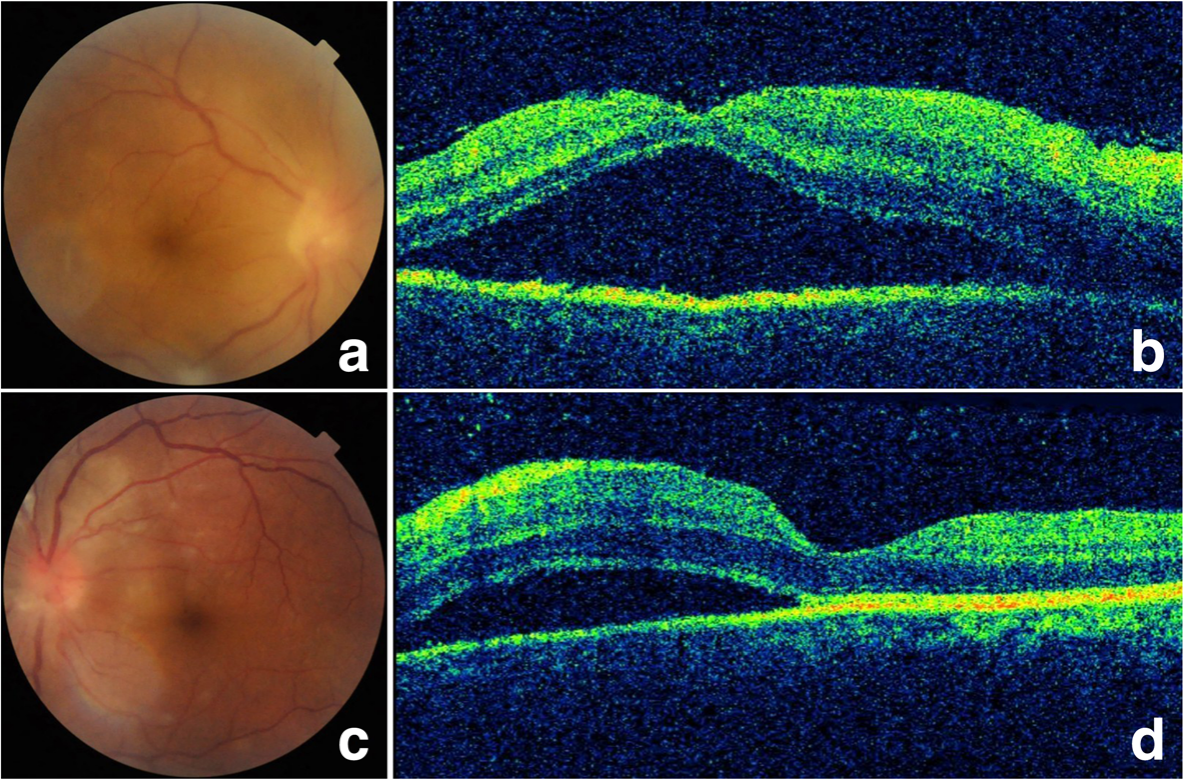
Fundus examination of case two (**a**, OD; **c**, OS) showed retina edema in the posterior area, retinal venous engorgement, and epiretinal membrane on the disk OU. The margin of optic disk was not clear OD. OCT (**b**, OD; **d**, OS) showed serous detachment of the macular and retinal neuroepithelium edema OU

A 37-year-old Chinese male presented on April 18, 2013, complaining blurred vision OU and recurrent abdominal pain for over a month. In March, he felt middle and upper abdominal pain, with abdominal distension, nausea, fatigue and profuse sweating. A few days later, he began to feel blurred vision OD, with water ripple feeling. He was diagnosed as necrotizing pancreatitis in local hospital. Abdominal pain relieved after treatment, but visual acuity of both eyes decreased. When the patient was referred to PUMCH, visual acuity was 20/125 OD and 20/32 OS. There were conjunctiva edema and congestion, gray-white KPs, anterior chamber cells and flare, and hazy vitreous OU. Fundus examination showed retina edema in the posterior area, retinal venous engorgement, and epiretinal membrane on the disk OU. The margin of optic disk was not clear OD (Fig. 3a, c). OCT showed serous detachment of the macular and retinal neuroepithelium edema OU (Fig. 3b, d). After using topical 1% Pred Forte and tropicamide phenylephrine eye drops for 2 weeks, anterior uveitis didn’t release. From April 28, the patient complained intermittent abdominal pain and fever. Serum amylopsin, lipase, transaminase and bilirubin were elevated. CT showed diffused enlargement of pancreas, edema of duodenal wall, thickening of gall blander wall. B ultrasound showed enlargement of the common bile duct. Visual acuity continued to decrease. On May 10, an emergency exploratory laparotomy operation was done. Unfortunately, the patient died of multiple organ failure 4 days later. Final histopathological diagnosis was gallbladder type II enteropathy-associated T-cell lymphoma (EATL).

## Discussion

Primary vitreoretinal lymphomas (PVRL) are the most common lymphoma of the eye, and most of them are extranodal, non-Hodgkin, diffuse large B-cell lymphomas [1]. Intraocular lymphomas of non-B-cell type are rare and represent only approximately 1–3% of all lymphoproliferative lesions in this site [3]. Most intraocular T-cell or NKT cell lymphomas represent a secondary manifestation of either a cutaneous or systemic lymphoma, and have been regarded as markers of poor prognosis [3–7].

Extranodal natural killer/T-cell lymphoma (NKTL) is an uncommon, aggressive neoplasm that is associated with EBV infection [8]. In addition, eyelid is another site that can be involved in cases of T cell lymphoma, and ocular symptoms could appear before systemic symptoms [9].

Cao et al. had reported a series of systemic metastatic retinal lymphoma (SMRL), including 9 B-SMRLs among 96 B-cell retina lymphomas (9.4%) and 3 T-SMRLs among 5 T-cell retinal lymphomas (60%). They found that SMRL and primary retinal lymphoma present with similar clinical manifestations, while systemic T-cell lymphoma invades the retina and vitreous more aggressively than systemic B-cell lymphoma [1]. Grace and colleagues had reviewed 29 cases of intraocular metastatic T-cell lymphomas that confirmed with ocular biopsy. Thirteen cases (44.8%) had a past history of a peripheral T-cell lymphoma. The most common presenting clinical features were vitritis (19/29, 65.5%) and non-granulomatous anterior uveitis (13/29, 44.8%) [8].

Our case one is a 19-year-old female with pharynx T cell non-Hodgkin’s lymphoma. NKTL especially that involves the ocular is generally a rapidly progressing disease, with short survival times from diagnosis despite standard therapy [10–13]. Persistent EBV infection of T or NK cells defines a distinct disease entity. It is important to consider lymphoma development as a possible event in patients with chronic EBV-associated liver disease [14]. Fortunately, the young girl responded well to chemotherapy, and has been survival for 10 years since presenting symptoms.

Our case two is a 37-year-old male complaining bilateral blurred vision and recurrent abdominal pain. Final diagnosis was gallbladder type II EATL. Unfortunately, 26 days after presenting to Ophthalmology department, the patient died of multiple organ failure. To the best of our knowledge, it is the first presentation of gallbladder type II EATL metastasizing to the eye. EATL is a lymphoma arising from intraepithelial T cells. The current WHO classification recognizes two variants, denoted type I and type II, and the latter constitutes 10–20% of cases of EATL [15]. Mudhar et al. had reported a 47-year-old man with small bowel EATL presented 2 years after chemotherapy because of floaters in both eyes. Fundoscopy showed bilateral vitritis, retina vasculitic changes and intraretinal haemorrhage. Right eye diagnostic vitrectomy confirmed metastatic EATL type II in the vitreous. The patient subsequently developed brain metastases with rapid neurological deterioration [16]. Our patient was similar to this case, but the progression of disease was so rapid that there was no time to do further examination and treatment after diagnosis.

The gold standard for diagnosing intraocular lymphoma remains cytopathologic examination of the ocular specimen. Critical adjunctive studies may include flow cytometry, immunophenotyping and molecular analyses [8]. The limitation of our two cases is lack of vitreous or aqueous humor samples to confirm lymphoma cells in the eye. There are three points support the diagnosis of systemic metastatic ocular T cell lymphoma: (1) Patients presented with systemic symptoms including fever, fatigue and abdominal pain. Diagnosis of uveitis were definite, but steroid and immunosuppressant therapy were not effective and inflammation was persistent. (2) Lesion in other sites were found and T cell lymphoma was confirmed by immunohistochemistry. (3) In case one, uveitis relieved and vision was stable after chemotherapy. It’s worth noting that in some cases of T cell lymphoma, the earliest symptoms occurring in the eye region without any systemic symptoms. A recent published case report of a 19 year old male of subcutaneous panniculitis-like T-cell lymphoma (SPTCL) presented with sudden eyelid swelling complicated by visual deterioration, and systemic symptoms appeared after the temporary improvement of symptoms by steroid administration. In such cases, timely diagnosis is more difficult [9].

## Conclusions

Intraocular involvement of systemic T-cell lymphomas are uncommon and have been broadly regarded as markers of poor prognosis. A diagnosis of lymphoma has to be suspected when patients have systemic manifestations including fever, fatigue, abdominal pain, EBV-associated liver disease, et al., and uveitis fails to respond to steroid therapy.

## Abbreviations

EATL: Enteropathy-associated T-cell lymphoma
NKTL: Natural killer/T-cell lymphoma
OD: Right eye
OS: Left eye
OU: Both eyes
PVRL: Primary vitreoretinal lymphoma
SMRL: Systemic metastatic retinal lymphoma
